# Is Anlotinib and Radiotherapy Combination Effective for Non-Small-Cell Lung Cancer with Brain Metastases? A Systematic Scoping Review and Meta-Analysis

**DOI:** 10.3390/ph18070974

**Published:** 2025-06-28

**Authors:** Helal F. Hetta, Mostafa A. Sayed Ali, Saleh F. Alqifari, Hoda A. Salem, Khulood A. Qasem, Fawaz E. Alanazi, Amirah Alhowiti, Amirah M. Alatawi, Hyder Mirghani, Tariq Alrasheed, Salwa Q. Bukhari, Khalid A. Almazyad, Sultan A. Alhumaid, Noura H. Abd Ellah, Hashim M. Aljohani, Yasmin N. Ramadan, Reem Sayad

**Affiliations:** 1 Division of Microbiology, Immunology and Biotechnology, Department of Natural Products and Alternative Medicine, Faculty of Pharmacy, University of Tabuk, Tabuk 71491, Saudi Arabia; 2 Department of Pharmacy Practice, Faculty of Pharmacy, University of Tabuk, Tabuk 71491, Saudi Arabia; ma-ali@ut.edu.sa (M.A.S.A.); salqifari@ut.edu.sa (S.F.A.); hsalem@ut.edu.sa (H.A.S.); 3 College of Medicine, Sulaiman Alrajhi University, Al Bukairiyah 52726, Saudi Arabia; dr.khulood@hazaea.com; 4 Department of Pharmacology and Toxicology, Faculty of Pharmacy, University of Tabuk, Tabuk 71491, Saudi Arabia; falanazi@ut.edu.sa; 5 Department of Family and Community Medicine, Faculty of Medicine, University of Tabuk, Tabuk 71491, Saudi Arabia; aalhowiti@ut.edu.sa (A.A.); am.alatawi@ut.edu.sa (A.M.A.); 6 Department of Internal Medicine, Faculty of Medicine, University of Tabuk, Tabuk 71491, Saudi Arabia; h.mirghani@ut.edu.sa (H.M.); talrasheed@ut.edu.sa (T.A.); 7 Diagnostic Radiology Department, Faculty of Medicine, University of Tabuk, P.O. Box 741, Tabuk 71491, Saudi Arabia; s.bukhari@ut.edu.sa; 8 Department of Adult Medical Oncology, King Abdulaziz Medical City, Riyadh 14611, Saudi Arabia; almazyadkh@mngha.med.sa; 9 Department of Family Medicine, King Salman Armed Forces Hospital, Tabuk 47512, Saudi Arabia; suaalhumaid@moh.gov.sa; 10 Department of Pharmaceutics and Pharmaceutical Technology, Faculty of Pharmacy, Badr University in Assiut, Naser City 2014101, Egypt; nora.1512@aun.edu.eg; 11 Department of Pharmaceutics, Faculty of Pharmacy, Assiut University, Assiut 71515, Egypt; 12 Department of Clinical Laboratory Sciences, College of Applied Medical Sciences, Taibah University, Madina 41477, Saudi Arabia; hsnani@taibahu.edu.sa; 13 Department of Pathology and Laboratory Medicine, College of Medicine, University of Cincinnati, Cincinnati, OH 45221, USA; 14 Department of Microbiology and Immunology, Faculty of Pharmacy, Assiut University, Assiut 71515, Egypt; yasmine_mohamed@pharm.aun.edu.eg; 15 Department of Histology, Faculty of Medicine, Assiut University, Assiut 71515, Egypt; reem.17289806@med.aun.edu.eg

**Keywords:** anlotinib, novel multi-targeting tyrosine kinase inhibitor, combined non-small-cell lung cancer, NSCLC, brain metastasis, radiotherapy, stereotactic radiotherapy, SRS, whole-brain radiotherapy, WBRT, cranial radiotherapy, CRT

## Abstract

**Background/Objectives**: Non-small-cell lung cancer (NSCLC) frequently metastasizes to the brain, significantly impacting patient prognosis and quality of life. Anlotinib, a novel tyrosine kinase inhibitor, has shown promise in treating NSCLC with brain metastasis. So, we aimed to evaluate the clinical efficacy of anlotinib and various types of radiotherapy combinations used to treat NSCLC patients with brain metastasis regarding overall survival and the treatment of internal and external lesions. **Methods**: A comprehensive literature search was conducted in the databases PubMed, Scopus, WoS, MedLine, and Cochrane Library up to April 2024. Studies assessing the efficacy of anlotinib combined with whole-brain radiotherapy (WBRT), stereotactic radiosurgery (SRS), or other radiotherapy modalities in NSCLC patients with brain metastasis were included. The primary outcomes were (a) the efficacy of anlotinib and radiotherapy on the intracranial lesions and OS and (b) the effectiveness of combined anlotinib and radiotherapy versus radiotherapy alone in NSCLC patients with brain metastasis. The secondary outcome was the efficacy of anlotinib and radiotherapy on extracranial progression. We used a combination of keywords and MeSH terms including ‘non-small cell lung cancer’ OR ‘NSCLC’, ‘brain metastases’, ‘anlotinib’, ‘radiotherapy’, ‘radiation therapy’, and ‘combined treatment’, among others. Boolean operators (AND, OR) were applied as appropriate to optimize the search strategy across databases. **Results**: Nine studies met the inclusion criteria, comprising 210 patients in the combination group and 228 patients in the radiotherapy alone group. The combination of anlotinib with radiotherapy showed a significant improvement in iPFS compared to radiotherapy alone, with a pooled risk ratio (RR) for iORR of 1.18 (95% CI: 1.00–1.39) and a pooled SMD for OS of 0.03 (95% CI: −0.29, 0.36). Radiotherapy combined with anlotinib also demonstrated enhanced intracranial and extracranial control rates. **Conclusions**: Anlotinib combined with radiotherapy, especially WBRT, offers a promising treatment strategy for NSCLC patients with brain metastasis, improving intracranial control. Further large-scale randomized controlled trials are needed to confirm these findings and optimize treatment protocols.

## 1. Introduction

Lung cancer remains one of the leading causes of cancer-related mortality in the USA [1]. It constitutes about 25% of cancer-related fatalities and results in more deaths than the combined totals of colon, breast, and prostate cancers. The American Cancer Society predicts approximately 131,880 deaths and 235,760 new cases of lung cancer in 2021 [2]. Non-small-cell lung cancer (NSCLC) accounts for approximately 85% of all lung cancer cases. At the initial diagnosis, 10% of NSCLC patients present with brain metastases (BMs), and 25% to 50% of these individuals would subsequently develop BMs with the progression of their disease [3]. The median overall survival (OS) for BM from NSCLC is two to three months [4]. A significant contributor to the morbidity and mortality associated with advanced NSCLC is intracranial metastasis that has proven resistant to normal systemic therapy. Whole-brain radiation therapy (WBRT), a treatment established historically, remains a prevalent option for addressing patients with multiple brain metastases with systemic therapies. This intervention is appropriate for patients who are not candidates for surgery or stereotactic radiosurgery (SRS). Patients receiving local treatment with neurosurgical resection or SRS have a limited number of brain metastases (≤3) or present only with symptoms [5]. Post-WBRT, the illness remission rate varies between 24% and 55%, while the median overall survival extends to 3 to 6 months [6]. WBRT is associated with somewhat inadequate management of pre-existing metastases; however, it demonstrates enhanced control of distant intracranial tumors and a notable occurrence of late neurocognitive adverse effects [7,8,9]. Nonetheless, there may be no discernible benefit when juxtaposing WBRT with supportive treatment alone, owing to the unfavorable overall survival observed in patients with multiple brain metastases receiving WBRT. A randomized controlled trial is investigating the efficacy of WBRT compared to supportive treatment alone in NSCLC with BM who are ineligible for SRS. This analysis indicates no significant improvement in overall quality of life or overall survival between the two groups [10]. Given that WBRT alone remains inadequate in delivering therapeutic efficacy for NSCLC patients with BM and poor prognoses, innovative treatment strategies are urgently required.

Neoangiogenesis is essential for bone marrow expansion, particularly under rapidly progressing circumstances. In patients with symptomatic multiple brain metastases who are resistant to prior conventional targeted therapies or exhibit no actionable genetic alterations, the combination of WBRT and antiangiogenic inhibitors may prove beneficial. In patients with unresectable BM from solid tumors, the REBECA phase I trial has demonstrated the feasibility of combining conventional WBRT with bevacizumab and has yielded preliminary efficacy data [11].

Anlotinib is a novel oral antiangiogenic agent that functions by inhibiting key tyrosine kinase receptors, including FGFR1-4, PDGFR a/b, c-Kit, and FLT3, as well as the vascular endothelial growth factor (VEGF) receptors (1/2/3) [12]. Anlotinib demonstrates substantial efficacy in addressing intracranial lesions and may benefit NSCLC patients with brain metastases who have not adequately responded to at least second-line therapy, as indicated by a post hoc analysis of the AL-TER0303 research [13]. These findings indicate that WBRT combined with anlotinib may be a valuable strategy for managing BM from NSCLC and necessitate further investigation.

Nonetheless, the efficacy of anlotinib in specific advanced lung cancer instances with BM is acknowledged, although cranial irradiation is presently considered the conventional treatment regimen for NSCLC patients lacking particular gene mutations or resistance to EGFR/ALK/ROS1-TKIs. It is attributed to its efficacy in swiftly alleviating central nervous system symptoms and prolonging patient survival duration [13,14]. CRT can enhance the permeability of the blood–brain barrier (BBB), potentially elevating the concentration of anlotinib in brain tissue [15]. In NSCLC patients lacking a particular gene mutation or exhibiting resistance to EGFR/ALK/ROS1-TKIs, the therapeutic efficacy of CRT combined with anlotinib may surpass that of CRT monotherapy.

In this systematic scoping review, we aimed to evaluate the clinical efficacy of anlotinib and various types of radiotherapy combinations used to treat NSCLC patients with brain metastasis in terms of OS and the treatment of internal and external lesions.

## 2. Methods

### 2.1. Information Sources and Search Strategy

Considering the PRISMA (Preferred Reporting Items for Systematic Reviews and Meta-Analyses) extension for scoping reviews, a systematic scoping review of clinical trials was created [16]. The protocol of this review was registered in PROSPERO. Protocol ID: CRD420251038438.

The databases SCOPUS, PubMed, Web of Science (WoS), Cochrane, and MedLine through WoS were examined up to 12 April 2024. The terminologies Anlotinib, a novel multi-targeting tyrosine kinase inhibitor, combined non-small-cell lung cancer, NSCLC, brain metastasis, radiotherapy, stereotactic radiotherapy, SRS, whole-brain radiotherapy, WBRT, and comparative clinical studies are the terms used to review interventional studies in all languages published up until April 2024. Then, we used the Boolean operators AND OR to search all databases. Details of the search strategy are mentioned in Supplementary Materials (Table S1).

### 2.2. Eligibility Criteria

Inclusion criteria: Only NSCLC patients who received anlotinib and radiotherapy for brain metastasis were included. All types of radiotherapy for brain metastasis were included, such as whole-brain radiotherapy (WBRT), stereotactic body radiotherapy (SBRT), stereotactic radiosurgery (SRS), or cranial radiotherapy (CRT). Prospective or retrospective trials were incorporated. Moreover, we included conference abstracts that published results from clinical trials that met our inclusion criteria. There was no restriction according to the language of the published trials.

Exclusion criteria: Experimental studies performed in vitro, animal studies, case reports, and case series were not included.

### 2.3. Research Questions

This systematic scoping review aims to respond to the following questions. In NSCLC patients with brain metastasis, (a) what is the efficacy of anlotinib and radiotherapy on intracranial lesions and OS as primary outcomes? (b) Is there a difference in efficacy between combined anlotinib and radiotherapy and radiotherapy alone? (c) What is the efficacy of combined anlotinib and radiotherapy on extracranial progression as a secondary outcome?

### 2.4. Trial Selection

Upon reviewing the abstracts and whole texts, specific keywords motivated both researchers to select the studies. The two researchers employed the inclusion criteria to evaluate the trials. Subsequently, each abstract and full-text was downloaded and assessed independently according to the predetermined inclusion criteria. In instances of conflict among the researchers, the third author evaluated the study’s acceptability.

### 2.5. Data Extraction

Two authors independently assessed and appraised each full text that satisfied the inclusion criteria for this systematic scoping review. Each investigator independently constructed a table encompassing the most critical details from the selected trials, and the results were subsequently compared. The table that summarizes the included studies lists the names of the authors, the publication year, the study design, the setting of the clinical trial, the date (period of the study), and the arm(s) of the clinical trial(s). The baseline characteristics include the arm(s) of the clinical trial(s), the sequence of follow-up, the regimen of treatment, sample size, median age, the percentage of the male population, and the pathological types of NSCLC. The baseline characteristics of the included studies are summarized in Table 1.

The clinical efficacy of the treatment in terms of the remission rate of OS, the effective rate of intracranial lesion treatment, and the effectiveness of extracranial progression.

### 2.6. Outcome Measures

The primary outcomes are (a) the efficacy of anlotinib and radiotherapy on the intracranial lesions and OS, and (b) the effectiveness of combined anlotinib and radiotherapy versus radiotherapy alone in NSCLC patients with brain metastasis.

The secondary outcomes is the efficacy of anlotinib and radiotherapy on extracranial progression.

The effective rate of overall intracranial lesion treatment was presented in the forms of complete remission (CR), partial remission (PR), stable disease (SD), progressive disease (PD), intracranial progression-free survival (iPFS), intracranial objective response rate (iORR), and intracranial disease control rate (iDCR). The effectiveness of extracranial progression was presented in the forms of eORR, eDCR, and ePFS.

### 2.7. Quality Assessment

The quality of the included studies was assessed by two authors independently using an instrument previously validated and comprising 16 criteria [26]. Each study had to be given a score by the authors of this review on a scale from 0 to 3 for each of the criteria. When the study authors did not provide enough information to reach a result about a point, they received a score of zero for that item. A value of three was assigned when the evaluated item’s level of certainty was provided. A value of two was taken as the default when the data was unclear. When these requirements were met, the body of evidence’s overall result was expressed as a percentage of the maximum score of 100%.

### 2.8. Data Synthesis

The statistical analysis was conducted using Open Meta-Analyst software version 1.15.14, as per the single-armed analysis [27,28]. In a random-effects model with a 95% confidence interval (CI), dichotomous and continuous variables were aggregated as an untransformed percentage (PR) and mean difference (MD), respectively. Forest plot graphs were employed to assess heterogeneity, with the extent of heterogeneity assessed by chi-square and I-square tests. A chi-square test with *p* < 0.1 and I^2^ tests > 50% demonstrated substantial variability among trials [29]. The endpoints were considered statistically significant when the *p*-value was below 0.05.

Utilizing the double-armed technique, we examined the data with Review Manager (RevMan) version 5.4. We conducted a comparison of anlotinib in conjunction with radiation to radiotherapy alone. Continuous data were evaluated as a standardized mean difference (SMD) due to the inclusion of studies employing several radiation modalities, including WBRT, SRS, SBRT, and CRT, utilizing the inverse variance approach and a 95% confidence interval (CI). Dichotomous data were evaluated as a risk ratio (RR) with the Mantel–Haenszel method with a 95% confidence interval (CI). A *p*-value of less than 0.05 indicated a significant difference. Heterogeneity was evaluated using forest plot graphs, I-squared (I^2^), and Chi-square (χ^2^) tests, deemed significant if I^2^ > 50% and the P-value of χ^2^ < 0.01. A random model was employed for all outcomes due to the variability in the study designs included. In cases with high heterogeneity, we conducted a leave-one-out test to mitigate the heterogeneity.

## 3. Results

### 3.1. Study Selection

**Figure 1:**
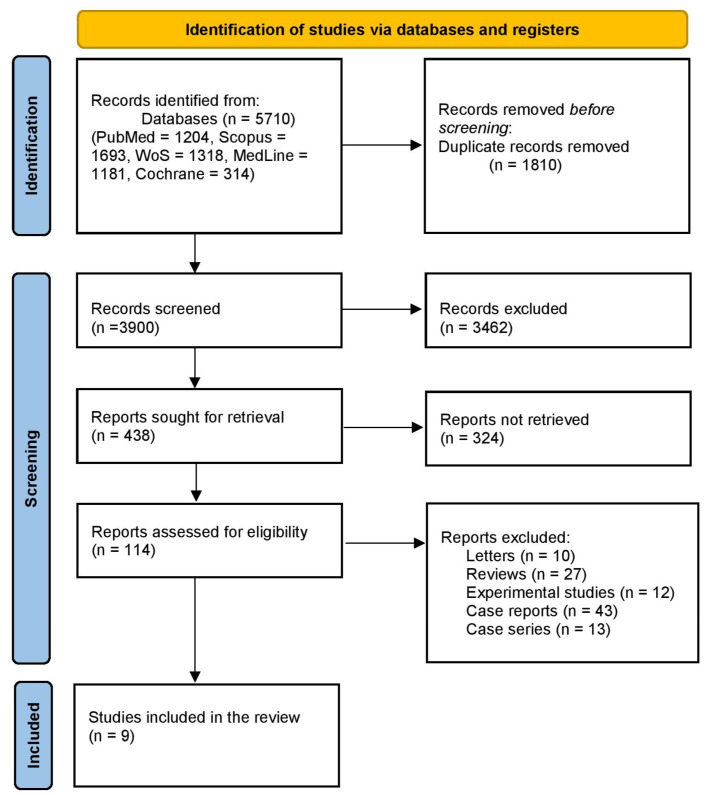
PRISMA flow diagram of included studies.

We initially retrieved 5710 studies, with 1810 excluded as duplicates, leaving 3900 for title and abstract screening. From these, 3462 were excluded, and 429 were further excluded after full-text review, resulting in 9 clinical trials included in the systematic scoping review (Figure 1).

#### Study Characteristics

Table 1 presents the characteristics of the included studies. They were published between 2020 and 2023. All of them are retrospective analyses except three studies, which are prospective single-armed phase II trials [19,24,25]. Three studies of the retrospective analysis compared the efficacy of WBRT alone to that of anlotinib combined with WBRT [17,18,22], two studies compared SRS alone to combined anlotinib and SRS [20,23], and one compared CRT to combined anlotinib and CRT [21] in NSCLC patients with brain metastasis. All studies were performed in China; three studies were at Jiangsu Cancer Hospital [18,22,24], two studies at Shaoxing People’s Hospital [17,19], two at Peking University Third Hospital [20,23], one at Hospital of Bengbu Medical College [21], and the hospital of one study was not mentioned [24].

The sample size ranged from 10 to 34 in the intervention group (combined anlotinib and radiotherapy) and 21 to 70 in the comparator group (radiotherapy alone). The median age of the intervention group ranged from 57.5 to 63.5 years old and that of the comparator group ranged from 55 to 60.5 years old. Adenocarcinoma was the most common pathological type reported in both groups, 117 (55.7%) vs. 112 (49.1%). Most studies used a brain MRI as a follow-up investigation method to assess the progression of the disease and detect any complications. The baseline characteristics of the patients are mentioned in Table 2.

### 3.2. Quality Assessment

All studies examined in this systematic review satisfied at least 72.9% of the specified quality standards [19], so they are classified as studies of good quality. Nonetheless, there is significant heterogeneity in the included studies, primarily due to the treatments taken into account in each study and secondly due to the different study designs of the included studies. The quality of four studies from the nine included studies was not assessed because they are conference abstracts only. Details of quality assessment are mentioned in Table 3.

### 3.3. Primary and Secondary Outcomes

#### 3.3.1. Overall Survival (OS)

**Figure 2:**
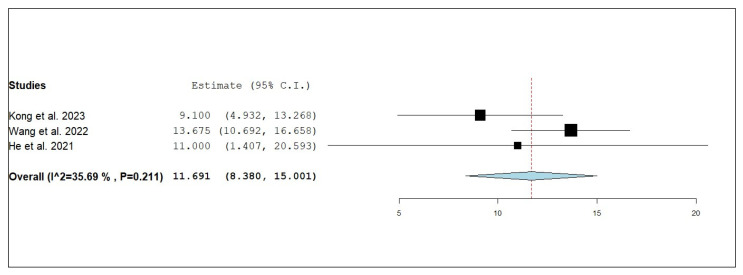
Single-arm meta-analysis of overall survival (OS) of NSCLC patients with brain metastasis who received anlotinib and radiotherapy [18,20,21].

According to the single-armed meta-analysis, three studies reported OS [18,20,21] and showed that the pooled mean OS was 11.691 months (95% CI, 8.38–15.001), with homogenous data (I^2^ = 35.69%; *p* = 0.211) (Figure 2).

**Figure 3:**
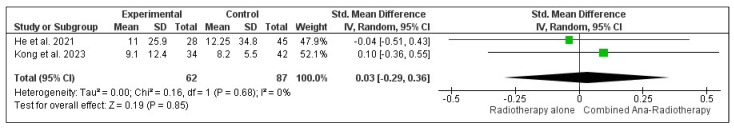
Double-arm meta-analysis of overall survival (OS) of NSCLC patients with brain metastasis who received combined anlotinib and radiotherapy in comparison to radiotherapy alone [18,21].

According to a double-armed meta-analysis, two studies reported OS in NSCLC patients with brain metastasis who received combined anlotinib and radiotherapy in comparison to radiotherapy alone [18,21]. There was no statistically significant difference in OS between combined therapy and radiotherapy alone (SMD = 0.03; *p* = 0.85; 95% CI, −0.29, 0.36), with homogenous data (I^2^ = 0%; *p* = 0.68) (Figure 3).

#### 3.3.2. Internal Progression-Free Survival (iPFS)

**Figure 4:**
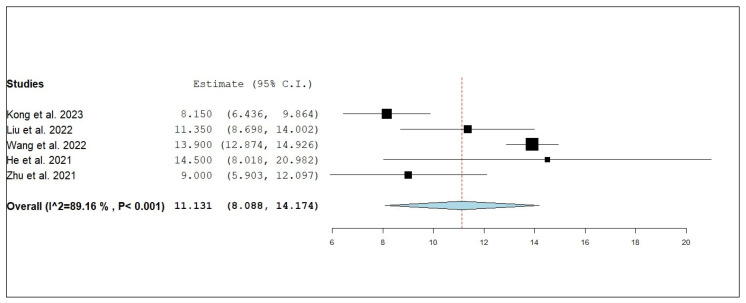
Single-arm meta-analysis of internal progression-free survival (iPFS) of NSCLC patients with brain metastasis who received anlotinib and radiotherapy [18,19,20,21,24].

**Figure 5:**
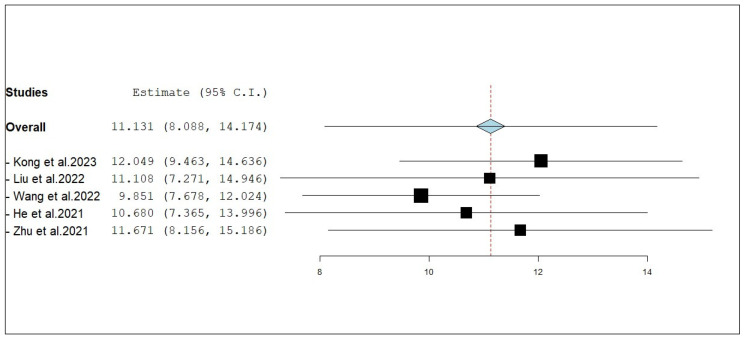
Leave-one-out test of internal progression-free survival (iPFS) of NSCLC patients with brain metastasis who received anlotinib and radiotherapy [18,19,20,21,24].

According to the single-armed meta-analysis, five studies reported iPFS [18,19,20,21,24] and showed that the pooled mean iPFS was 11.131 months (95% CI, 8.088–14.174), with significant heterogeneity (I^2^ = 89.16%; *p* < 0.001) (Figure 4). A leave-one-out test was performed to address this. The analysis shows that the removal of any single study does not substantially alter the overall effect size or confidence interval, suggesting that the meta-analysis results are stable and not unduly influenced by any individual study. The individual estimates for each study, when excluded, ranged from 9.851 months [20] to 12.049 months [18], all with overlapping confidence intervals. These findings indicate a consistent benefit in iPFS for NSCLC patients with brain metastases receiving combined anlotinib and radiotherapy, and support the robustness of the pooled effect estimate (Figure 5).

**Figure 6:**
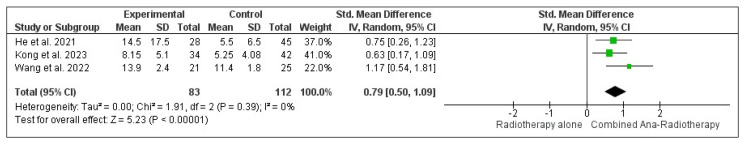
Double-arm meta-analysis of internal progression-free survival (iPFS) of NSCLC patients with brain metastasis who received combined anlotinib and radiotherapy in comparison to radiotherapy alone [18,19,21].

According to a double-armed meta-analysis, three studies reported iPFS in NSCLC patients with brain metastasis who received combined anlotinib and radiotherapy in comparison to radiotherapy alone [18,20,21]. A statistically significant improvement in iPFS with combined therapy (SMD= 0.79; *p* < 0.00001; 95% CI, 0.50, 1.09), with homogenous data (I^2^ = 0%; *p* = 0.39) (Figure 6).

#### 3.3.3. Internal Objective Response Rate (iORR)

**Figure 7:**
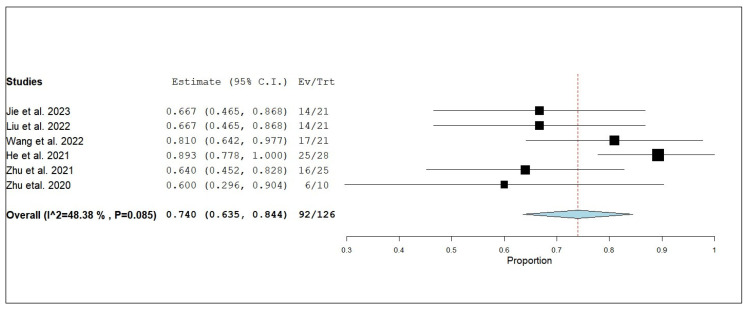
Single-arm meta-analysis of internal objective response rate (iORR) of NSCLC patients with brain metastasis who received anlotinib and radiotherapy [17,19,20,21,24,25].

**Figure 8:**
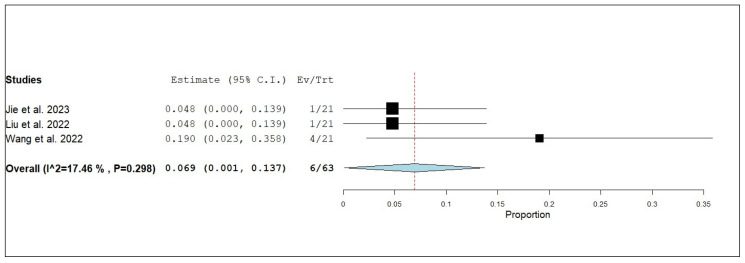
Single-arm meta-analysis of internal complete response (iCR) of NSCLC patients with brain metastasis who received anlotinib and radiotherapy [17,19,20].

**Figure 9:**
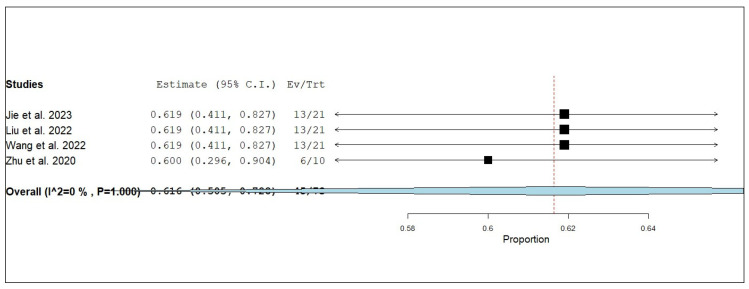
Single-arm meta-analysis of internal partial response (iPR) of NSCLC patients with brain metastasis who received anlotinib and radiotherapy [17,19,20,25].

**Figure 10:**
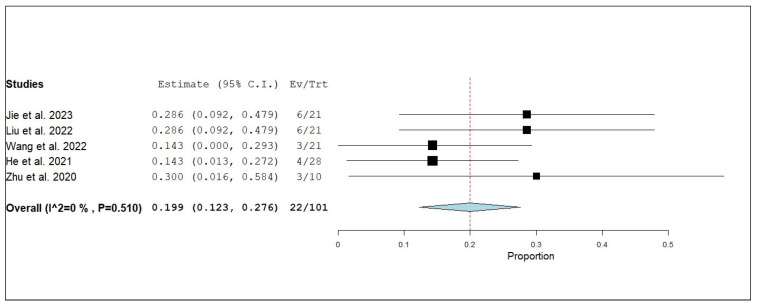
Single-arm meta-analysis of internal stable disease (iSD) of NSCLC patients with brain metastasis who received anlotinib and radiotherapy [17,19,20,21,25].

**Figure 11:**
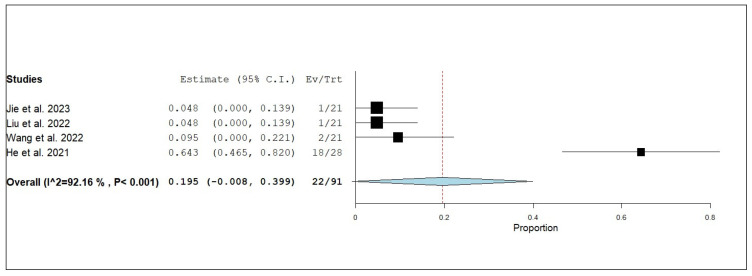
Single-arm meta-analysis of internal progression of the disease (iPD) of NSCLC patients with brain metastasis who received anlotinib and radiotherapy [17,19,20,21].

**Figure 12:**
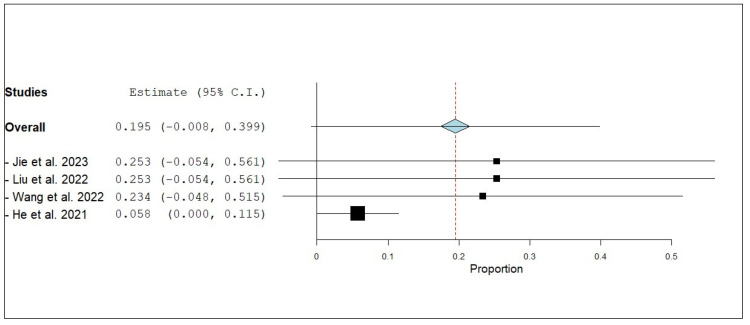
Leave-one-out test of internal progression of disease (iPD) of NSCLC patients with brain metastasis who received anlotinib and radiotherapy [17,19,20,21].

According to the single-armed meta-analysis, six studies reported iORR [17,19,20,21,24,25] and showed that the proportion of iORR was 0.740 (95% CI, 0.635–0.844), with homogenous data (I^2^ = 48.38%; *p* = 0.085) (Figure 7). Three studies reported the internal complete response (iCR) [17,19,20] and showed that the proportion of iCR was 0.069 (95% CI, 0.001–0.137), with homogeneous data (I^2^ = 17.46%; *p* = 0.298) (Figure 8). Four studies reported the internal partial response (iPR) [17,19,20,25] and showed that the proportion of iPR was 0.616 (95% CI, 0.505–0.728), with homogeneous data (I^2^ = 0%; *p* = 1) (Figure 9). Five studies reported the internal stable disease (iSD) [17,19,20,21,25] and showed that the proportion of iSD is 0.199 (95% CI, 0.123–0.276), with homogeneous data (I^2^ = 0%; *p* = 0.510) (Figure 10). Four studies reported the internal progression of the disease (iPD) [17,19,20,21] and showed that the proportion of iPD was 0.195 (95% CI, 0.008–0.399), with significant heterogeneity (I^2^ = 92.16%; *p* = 0.001) (Figure 11). A leave-one-out test was performed to address this. When each study was sequentially excluded, the resulting estimates remained generally consistent with the overall finding. The iPD estimates ranged from 0.058 (95% CI: 0.000 to 0.115; [21]) to 0.253 (95% CI: −0.054 to 0.561; [17,19]), with overlapping confidence intervals. Notably, the estimate from [21] had the narrowest confidence interval and was the only one that did not cross zero. Overall, the analysis indicates that no single study disproportionately influenced the pooled result, supporting the robustness and reliability of the iPD estimate in patients receiving the combination therapy (Figure 12).

**Figure 13:**
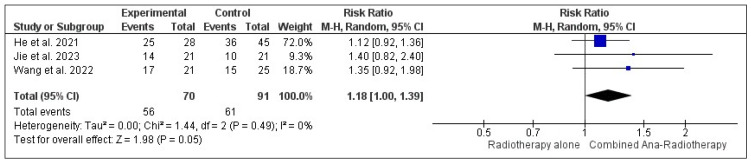
Double-arm meta-analysis of internal objective response rate (iORR) of NSCLC patients with brain metastasis who received combined anlotinib and radiotherapy in comparison to radiotherapy alone [17,20,21].

According to a double-armed meta-analysis, three studies reported iORR in NSCLC patients with brain metastasis who received combined anlotinib and radiotherapy in comparison to radiotherapy alone [17,20,21]. There was borderline statistical significance in the iORR risk ratio between groups (RR = 1.18; *p* = 0.05; 95% CI, 1.00, 1.39), with homogenous data (I^2^ = 0%; *p* = 0.49) (Figure 13).

#### 3.3.4. Internal Disease Control Rate (iDCR)

**Figure 14:**
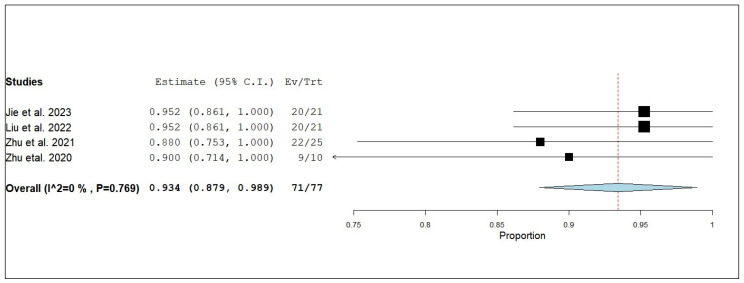
Single-arm meta-analysis of internal disease control rate (iDCR) of NSCLC patients with brain metastasis who received analotinib and radiotherapy [17,19,24,25].

According to the single-armed meta-analysis, four studies reported iDCR [17,19,24,25] and showed that the proportion of iDCR in patients who received combined radiotherapy and anlotinib was 0.934 (95% CI, 0.879–0.989), with homogeneous data (I^2^ = 0%; *p* = 0.769) (Figure 14).

#### 3.3.5. External Progression-Free Survival (ePFS)

**Figure 15:**
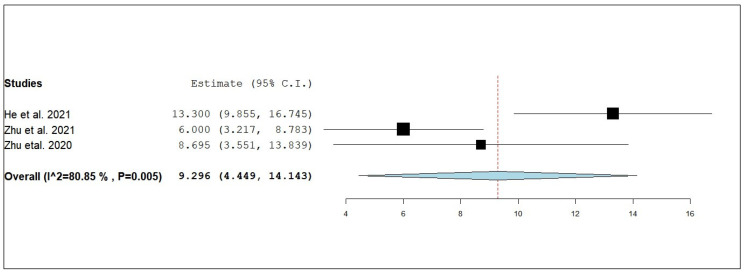
Single-arm meta-analysis of external progression-free survival (ePFS) of NSCLC patients with brain metastasis who received anlotinib and radiotherapy [21,24,25].

**Figure 16:**
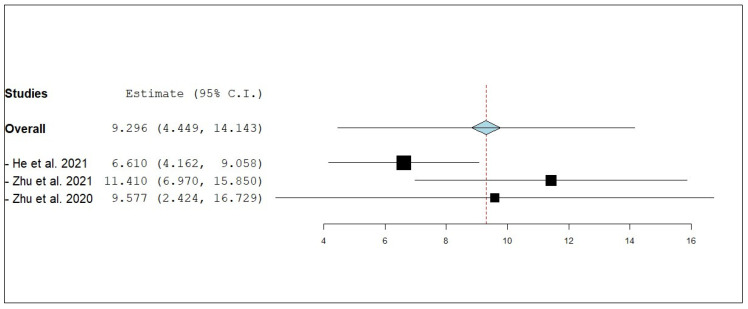
Leave-one-out test of external progression-free survival (ePFS) of NSCLC patients with brain metastasis who received anlotinib and radiotherapy [21,24,25].

According to the single-armed meta-analysis, three studies reported ePFS [21,24,25] and showed that the pooled mean of ePFS in patients who received combined radiotherapy and anlotinib was 9.296 months (95% CI, 4.449–14.143), with significant heterogeneity (I^2^ = 80.85%; *p* = 0.005) (Figure 15). A leave-one-out test was performed to address this. The leave-one-out sensitivity analysis demonstrated that the overall pooled estimate for ePFS remained consistent when each study was excluded sequentially, indicating that no single study unduly influenced the results. Specifically, the pooled ePFS estimate was 6.610 months (95% CI: 4.162–9.058) when [21] was omitted, 11.410 months (95% CI: 6.970–15.850) when [24] was excluded, and 9.577 months (95% CI: 2.424–16.729) upon the exclusion of [25]. The overlapping confidence intervals and consistent direction of effect suggest a stable and robust pooled outcome, reinforcing the potential benefit of anlotinib combined with radiotherapy in prolonging ePFS among NSCLC patients with brain metastases (Figure 16).

#### 3.3.6. External Objective Response Rate (eORR)

**Figure 17:**
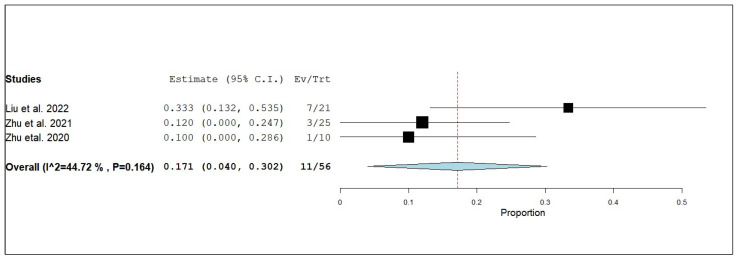
Single-arm meta-analysis of external objective response rate (eORR) of NSCLC patients with brain metastasis who received anlotinib and radiotherapy [17,24,25].

According to the single-armed meta-analysis, three studies reported eORR [19,24,25] and showed that the proportion of eORR in patients who received combined radiotherapy and anlotinib was 0.171 (95% CI, 0.040–0.302), with homogeneous data (I^2^ = 44.72%; *p* = 0.164) (Figure 17).

#### 3.3.7. External Disease Control Rate (eDCR)

**Figure 18:**
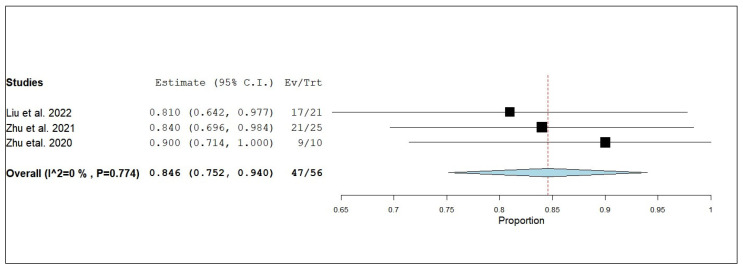
Single-arm meta-analysis of external disease control rate (eDCR) of NSCLC patients with brain metastasis who received anlotinib and radiotherapy [17,24,25].

According to the single-armed meta-analysis, three studies reported eDCR [19,24,25] and showed that the proportion of eDCR in patients who received combined radiotherapy and anlotinib was 0.846 (95% CI, 0.752–0.940), with homogeneous data (I^2^ = 0%; *p* = 0.774) (Figure 18).

## 4. Discussion

Non-small-cell lung cancer frequently metastasizes to the brain, significantly impacting patient prognosis and quality of life [30]. Anlotinib, a novel tyrosine kinase inhibitor, has shown promise in treating NSCLC with brain metastasis [31,32]. This review analyzed nine clinical trials to evaluate the efficacy of combining anlotinib with various forms of radiotherapy in NSCLC patients with brain metastasis. The studies generally showed a significant improvement in iPFS and iDCR with the combined treatment compared to radiotherapy alone. However, OS showed no statistically significant difference due to the very small sample size. The findings suggest that anlotinib combined with radiotherapy may offer some benefits in OS, iPFS, and ePFS. The reported findings for iDCR, eDCR, and eORR are based on single-arm analyses and, therefore, do not permit definitive conclusions about the comparative effectiveness of the combination therapy. Additionally, the improvement in iORR was only borderline statistically significant (*p* = 0.05). The observed response rates are potentially promising, but there is a need for further controlled studies to confirm the efficacy of the combination treatment in improving disease control and response rates.

The quality of the studies varied, and there was considerable heterogeneity, particularly in progression-free survival outcomes. This significant heterogeneity is due to the various study designs, and the treatments taken into account in each study.

Targeted medications, such as ALK inhibitors or EGFR-TKIs, have demonstrated intracranial efficacy, supporting their use as first-line therapy in NSCLC cases that have BMs with rearrangement of ALK or a mutation of EGFR, either in conjunction with or apart from cranial radiation therapy [33,34,35]. When surgery or SRS are not advised, the only modestly effective treatments left to control progressive intracranial disease after resistance to these targeted drugs are WBRT and chemotherapy, if they have not received them previously. Limited therapy options are available for dispersed BMs for patients lacking targetable mutations. Patients who have resistance to standardized systemic treatment may suffer from an aggressive intracranial disease at a late stage. This disease often progresses quickly and is characterized by radiologically detected pseudopalisading necrosis and/or peritumoral vasogenic edema, along with worsening clinical symptoms and poor performance status. These collective imaging characteristics most likely point to a more significant reliance of these malignancies on VEGF signaling. Additionally, preclinical research has shown that radiation and antiangiogenic therapy work synergistically; as a result, WBRT may be more effective when paired with therapies that target the pathways involved in tumor and vascular growth [36]. WBRT may also improve the penetration of some medications, which could increase their efficacy [37]. When anlotinib is used as a consolidation drug, it cures or stabilizes both extracranial disease and potentially subsequent cerebral micrometastatic disease simultaneously. WBRT plus concurrent anlotinib is more efficacious in managing intracranial disease. The previously described explanations could easily account for the anlotinib group’s prolonged iPFS in our systematic scoping review. Therefore, adding anlotinib to WBRT could be a tempting way to combat intracranial illness that worsens over time. Anlotinib plus radiotherapy has been shown in prior clinical trials to significantly enhance CNS-PFS, ePFS, OS, ORR, and DCR [20].

### 4.1. Strengths and Limitations

This is the first meta-analysis to assess the efficacy of anlotinib and various types of radiotherapy combinations in patients with advanced NSCLC with brain metastasis. An anlotinib plus radiotherapy combination could be a promising treatment in these patients.

Despite these promising results, some limitations must be acknowledged. There is a lack of RCTs that assess the efficacy of anlotinib and various types of radiotherapy in advanced NSCLC with brain metastasis. The generalizability of our results is limited because all of the included studies were conducted in China. Secondly, due to the small population size, the results of this study may be affected to some extent. The significant heterogeneity also forms one of the limitations of our study.

### 4.2. Recommendations

Future research should consider conducting randomized controlled trials that assess the efficacy of anlotinib and radiotherapy combination in advanced NSCLC patients with brain metastasis, especially after the failure of radiotherapy alone to control the disease. They should also consider performing controlled trials with a larger sample size to statistically detect the effect of the combination intervention. We also need to conduct studies that focus on adverse drug reactions to provide a clearer understanding of their impact. If there is a future meta-analysis, it should take a holistic approach by considering a combination of studies with larger sample sizes and clearer insights into adverse drug reactions to mitigate the limitations and enhance the overall quality of the analysis. Further high-quality research is needed to confirm these results.

## 5. Conclusions

This systematic scoping review evaluated the efficacy of combining anlotinib with various forms of radiotherapy in NSCLC patients with brain metastasis. The studies generally showed a trend towards improved iPFS and iDCR with the combined treatment compared to radiotherapy alone. The findings suggest that anlotinib combined with radiotherapy may offer some benefits in disease progression control, although further high-quality research is needed to confirm these results and assess their impact on overall survival.

## Figures and Tables

**Table 1:** Summary of included studies.

Authors	Year	Study Design	Setting	Date (Period of the Study)	Group(s)
Jie et al., 2023 [17]	2023	Retrospective Analysis	Shaoxing People’s Hospital, China	From March 2018 to March 2022	Combined anlotinib and WBRT
					Single WBRT group
Kong et al., 2023 [18]	2023	Retrospective Control Study	Jiangsu Cancer Hospital, Nanjing, China	From 2019 to 2021	Concurrent WBRT and anlotinib
					WBRT alone or in combination with other systemic agents
Liu et al., 2022 [19]	2022	Prospective Single-Arm, Phase II Study	Shaoxing People’s Hospital, Zhejiang Province, China	From April 2019 to March 2021	Combined WBRT and anlotinib
Wang et al., 2022 [20]	2022	Retrospective Cohort	Peking University Third Hospital, Beijing, China	From October 2017 to June 2019	Combined anlotinib and SRS
					Single SRS group
He et al., 2021 [21]	2021	Retrospective Control Study	Hospital of Bengbu Medical College, China	From September 2016 to June 2020	Combined anlotinib and CRT
					CRT alone
Kong et al., 2021 [22]	2021	Retrospective Control Study	Jiangsu Cancer Hospital, Nanjing, China	From 2019 to 2020	Concurrent WBRT and anlotinib
					WBRT alone or in combination with other systemic agents
Zhuang et al., 2021 [23]	2021	Case–Control Study	Peking University Third Hospital, Beijing, China	From October 2017 to June 2019	Combined anlotinib and SRS
					Single SRS group
Zhu et al., 2021 [24]	2021	Phase II Clinical Study	Jiangsu Cancer Hospital, Nanjing, China	At data cut-off 25 Jan 2021	Combined anlotinib and WBRT
Zhu et al., 2020 [25]	2020	Open-Label, Single-Arm Phase II Trial	NA	At data cut-off 30 April 2020	Combined anlotinib and WBRT

SRS: stereotactic radiosurgery; SBRT: stereotactic body radiotherapy; CRT: cranial radiotherapy; LCRT: local cranial radiotherapy; IQR: interquartile range; PD: disease progression. NA: not assessed.

**Table 2:** Baseline characteristics of included patients.

Authors	Group(s)	Regimen of Treatment	Sequence of Follow-Up	Sample Size	Median Age (years) *	Male Population N (%)	Pathological Types N (%)
Jie et al., 2023 [17]	Combined anlotinib and WBRT	Oral anlotinib (12 mg/d, day 1 to day 14, 21 days per cycle) at the same time at the start of radiotherapy for two cycles, combined with 30 Gy/10 times (5 times/week).	Tumor response was assessed every 8 weeks using brain MRI until PD. All patients were followed up by a combination of telephone and outpatient visits.	21	12 pts (57.1%) were aged > 60 years	13 (61.9)	Adenocarcinoma 18 (85.7) Squamous cell carcinoma 3 (14.3)
	Single WBRT group	WBRT in the form of 30 Gy/10 times (5 times/week).		21	15 pts (71.4%) were aged > 60 years	14 (66.7)	Adenocarcinoma 17 (81.0) Squamous cell carcinoma 4 (19.0)
Kong et al., 2023 [18]	Concurrent WBRT and anlotinib	Anlotinib (8–12 mg daily for 14 days) orally per 3-week cycle, combined with WBRT 30 Gy in 10–12 daily fractions, ideally given over 2–2.5 weeks with a 6-MV linear accelerator with two parallel opposed fields.	Brain MRIs were used to evaluate the intracranial disease at baseline and during a follow-up period every 2–3 months. Median follow-up: 21 (IQR: 14–22) months	34	61	18 (53)	Adenocarcinoma 29 (85) Others 5 (15)
	WBRT alone or in combination with other systemic agents	WBRT was defined as 30 Gy in 10–12 daily fractions, ideally given over 2–2.5 weeks with a 6-MV linear accelerator with two parallel opposed fields.		42	60.5	20 (48)	Adenocarcinoma 37 (88) Others 5 (12)
Liu et al., 2022 [19]	Combined WBRT and anlotinib	WBRT (30Gy/10 f, 5 f/week) and anlotinib (12 mg/day, day 1–14 of 21 days per cycle, 2 cycles) until disease progression or treatment intolerance.	The median follow-up time was 10.37 (3.27–26.13) months.	21	12 pts (57.1%) were aged ≥ 60 years	13 (61.9)	Adenocarcinoma 18 (85.7) Squamous cell carcinoma 3 (14.3)
Wang et al., 2022 [20]	Combined anlotinib and SRS	CyberKnife SRS + Oral anlotinib (12 mg/d) were taken for 2 weeks, 1 week off, and 21 days	A routine brain MRI examination was performed 1.5 to 2 months after treatment and then the patients were reexamined once every 2 to 3 months.	21	61 (32, 74)	12 (57.14)	Adenocarcinoma 16 (76.19) Squamous cell carcinoma 4 (19.047) Undifferentiated carcinoma 1 (4.7)
	Single SRS group	CyberKnife SRS		25	55 (39, 71)	13 (52)	Adenocarcinoma 20 (80) Squamous cell carcinoma 4 (16) Undifferentiated carcinoma 1 (4)
He et al., 2021 [21]	Combined anlotinib and CRT	Anlotinib (8–12 mg daily for 14 days) orally and then 7 days off, combined with CRT treatment (6 MV X-ray)	Clinical follow-up was carried out every 3–6 months and included imaging, physical, and routine laboratory tests. Median follow-up 8 months	28	63.5 (30–75)	15 (53.57)	Adenocarcinoma 21 (75) Squamous carcinoma 7 (25)
	CRT alone	CRT treatment (6 MV X-rays) included WBRT, WBRT plus LCRT, or LCRT.		45	56 (41–73)	24 (53.33)	Adenocarcinoma 38 (84.44) Squamous carcinoma 7 (15.56)
Kong et al., 2021 [22]	Concurrent WBRT and anlotinib	Anlotinib (8–12 mg daily for 14 days) orally per 3-week cycle, combined with WBRT 30 Gy in 10–12 daily fractions, ideally given over 2–2.5 weeks with a 6-MV linear accelerator with two parallel opposed fields.	NA	26	NA	NA	NA
	WBRT alone or in combination with other systemic agents	WBRT was defined as 30 Gy in 10–12 daily fractions, ideally given over 2–2.5 weeks with a 6-MV linear accelerator with two parallel opposed fields.		70			
Zhuang et al., 2021 [23]	Combined anlotinib and SRS	CyberKnife SRS + Oral anlotinib 12 mg/d was taken for 2 weeks, 1 week off, 21 days.	NA	21	NA	NA	NA
	Single SRS group	CyberKnife SRS		25			
Zhu et al., 2021 [24]	Combined anlotinib and WBRT	Anlotinib (12 mg, QD, day 1 to 14 of a 21-day cycle) combined with WBRT (DT 30 Gy/12f), followed by maintenance therapy with anlotinib until disease progression or treatment intolerance.	NA	28	57.5	13 (46.4)	Adenocarcinoma 25 (89.3) EGFR mutation 6 (21.4)
Zhu et al., 2020 [25]	Combined anlotinib and WBRT	Anlotinib (12 mg, QD, day 1 to 14 of a 21-day cycle) combined with WBRT (DT 30 Gy/12 times).	NA	10	NA	NA	NA

* Age presented at median (interquartile range). NA: not assessed.

**Table 3:** Quality assessment of included studies.

Authors	A	B	C	D	E	F	G	H	I	J	K	L	M	N	O	P	Overall Quality
Jie et al., 2023 [17]	3	3	3	2	1	2	2	2	2	2	3	3	3	2	0	3	75
Kong et al., 2023 [18]	3	3	3	1	1	2	2	2	3	2	3	3	3	3	0	3	77.083
Liu et al., 2022 [19]	3	3	3	1	1	2	2	3	2	2	3	3	2	2	0	3	72.916
Wang et al., 2022 [20]	2	3	3	3	1	3	2	2	2	2	3	3	3	2	0	3	77.083
He et al., 2021 [21]	3	3	3	1	1	3	2	3	2	2	3	3	2	2	0	3	75

The quality of [22,23,24,25] was not assessed because they are conference abstracts only.

## Data Availability

Not applicable.

## Supplementary Materials

The following supporting information can be downloaded at: https://www.mdpi.com/article/10.3390/ph18070974/s1, Supplementary Table S1: Details of the full search strategies for each database.
