# Supplementary material for: Is Anlotinib and Radiotherapy Combination Effective for Non-Small-Cell Lung Cancer with Brain Metastases? A Systematic Scoping Review and Meta-Analysis
Source: Pharmaceuticals (Basel). 2025 Jun 28;18(7):974. doi: 10.3390/ph18070974 (PMC12298774; doi:10.3390/ph18070974)
Supplement: Supplementary file 1 [file pharmaceuticals-18-00974-s001.zip › Supplementary material S1.pdf]

Title:

Is Anlotinib and Radiotherapy Combination Effective for Non-Small Cell Lung Cancer with Brain Metastases? A Systematic Scoping Review and Meta-Analysis.

Table S1: Details of the search strategy.

Database	Search strategy	Results
Pubmed	((Anlotinib OR “receptor tyrosine kinase inhibitor” OR “Novel Targeted Drug” OR “novel multi-targeting tyrosine kinase inhibitor”) AND (Carcinoma, Non-Small-Cell Lung OR NSCLC OR Lung Carcinoma, Non-Small-Cell OR Lung Carcinomas, Non-Small-Cell OR Non-Small-Cell Lung Carcinomas OR Non-Small-Cell Lung Carcinoma OR Non Small Cell Lung Carcinoma OR Carcinoma, Non-Small Cell Lung OR Non-Small Cell Lung Carcinoma OR Non-Small Cell Lung Cancer OR Nonsmall Cell Lung Cancer))	1204
Scopus	((Anlotinib OR “receptor tyrosine kinase inhibitor” OR “Novel Targeted Drug” OR “novel multi-targeting tyrosine kinase inhibitor”) AND (Carcinoma, Non-Small-Cell Lung OR NSCLC OR Lung Carcinoma, Non-Small-Cell OR Lung Carcinomas, Non-Small-Cell OR Non-Small-Cell Lung Carcinomas OR Non-Small-Cell Lung Carcinoma OR Non Small Cell Lung Carcinoma OR Carcinoma, Non-Small Cell Lung OR Non-Small Cell Lung Carcinoma OR Non-Small Cell Lung Cancer OR Nonsmall Cell Lung Cancer))	1693
WoS	((Anlotinib OR “receptor tyrosine kinase inhibitor” OR “Novel Targeted Drug” OR “novel multi-targeting tyrosine kinase inhibitor”) AND (Carcinoma, Non-Small-Cell Lung OR NSCLC OR Lung Carcinoma, Non-Small-Cell OR Lung Carcinomas, Non-Small-Cell OR Non-Small-Cell Lung	1318

	Carcinomas OR Non-Small-Cell Lung Carcinoma OR Non Small Cell Lung Carcinoma OR Carcinoma, Non-Small Cell Lung OR Non-Small Cell Lung Carcinoma OR Non-Small Cell Lung Cancer OR Nonsmall Cell Lung Cancer)) (All Fields)	
MedLine	((Anlotinib OR “receptor tyrosine kinase inhibitor” OR “Novel Targeted Drug” OR “novel multi-targeting tyrosine kinase inhibitor”) AND (Carcinoma, Non-Small-Cell Lung OR NSCLC OR Lung Carcinoma, Non-Small-Cell OR Lung Carcinomas, Non-Small-Cell OR Non-Small-Cell Lung Carcinomas OR Non-Small-Cell Lung Carcinoma OR Non Small Cell Lung Carcinoma OR Carcinoma, Non-Small Cell Lung OR Non-Small Cell Lung Carcinoma OR Non-Small Cell Lung Cancer OR Nonsmall Cell Lung Cancer))	1181
Cochrane	((Anlotinib OR “receptor tyrosine kinase inhibitor” OR “Novel Targeted Drug” OR “novel multi-targeting tyrosine kinase inhibitor”) AND (Carcinoma, Non-Small-Cell Lung OR NSCLC OR Lung Carcinoma, Non-Small-Cell OR Lung Carcinomas, Non-Small-Cell OR Non-Small-Cell Lung Carcinomas OR Non-Small-Cell Lung Carcinoma OR Non Small Cell Lung Carcinoma OR Carcinoma, Non-Small Cell Lung OR Non-Small Cell Lung Carcinoma OR Non-Small Cell Lung Cancer OR Nonsmall Cell Lung Cancer))	314
